# Earthworm Coelomocytes and Coelomic Fluid: Innate Immunity, Toxicological Responses, and Research Applications

**DOI:** 10.3390/ani16121921

**Published:** 2026-06-21

**Authors:** Dora Bjedov, Lucija Sara Kovačić, Mirna Velki, Sandra Ečimović

**Affiliations:** Department of Biology, Josip Juraj Strossmayer University of Osijek, Cara Hadrijana 8/A, 31000 Osijek, Croatia; dora.bjedov@biologija.unios.hr (D.B.);

**Keywords:** earthworms, coelomocytes, coelomic fluid, innate immunity, ecotoxicology, biomarkers, lysenin, antimicrobial peptides, soil pollution, bioindicators

## Abstract

Earthworms possess a highly developed innate immune system based on the coordinated activity of coelomocytes and humoral factors present in the coelomic fluid. These immune components play a central role in host defence against pathogens, maintenance of physiological homeostasis, and adaptation to environmental stressors. Coelomocytes exhibit remarkable functional and morphological diversity, including participation in phagocytosis, encapsulation, extracellular trap formation, cytotoxic responses, wound healing, and regulation of oxidative and osmotic stress. In addition, coelomic fluid contains numerous biologically active molecules, such as lysenin, coelomic cytolytic factor 1, perforin, serine proteases, lysozyme, antimicrobial peptides, and pattern recognition receptors, which contribute to cellular and humoral immune responses. Recent studies have demonstrated that earthworm coelomocytes are highly sensitive to environmental pollutants, including heavy metals, pesticides, nanomaterials, and microplastics, highlighting their importance in ecotoxicological research and soil biomonitoring. Furthermore, antifungal, antimicrobial, anti-inflammatory, antipyretic, and cytotoxic activities associated with coelomocytes and coelomic fluid suggest promising applications in agriculture, biotechnology, and pharmaceutical research. This review summarises current knowledge regarding the classification, characteristics, immune functions, toxicological responses, and applied significance of earthworm coelomocytes and coelomic fluid, with particular emphasis on their role in environmental monitoring and potential biomedical applications.

## Simple Summary

Earthworm coelomocytes and coelomic fluid represent a complex innate immune system involved in host defence, maintenance of physiological homeostasis, and responses to environmental stress. Coelomocytes participate in numerous cellular immune mechanisms, including phagocytosis, brown body formation, extracellular trap formation, rejection of foreign tissue, and cytotoxic activity against tumour cells. In addition to cellular responses, humoral immunity is mediated by biologically active molecules present in coelomic fluid, such as lysenin, coelomic cytolytic factor 1, perforin, serine proteases, lysozyme, and antimicrobial peptides. Recent studies have demonstrated that earthworm coelomocytes are highly sensitive to pollutants, including heavy metals, pesticides, nanomaterials, and microplastics. Changes in coelomocyte viability, oxidative status, lysosomal stability, phagocytic activity, and fluorescence highlight their importance as sensitive biomarkers in ecotoxicological and environmental monitoring studies. Furthermore, the antimicrobial, antifungal, anti-inflammatory, and cytotoxic properties of coelomic fluid components suggest considerable potential for applications in biotechnology, agriculture, and pharmaceutical research. Despite significant progress, important gaps remain regarding coelomocyte classification, signalling pathways, and mechanisms underlying immune and toxicological responses. Future research integrating molecular, physiological, and ecological approaches will improve our understanding of earthworm immunity and strengthen the application of coelomocytes in environmental biomonitoring and biomedical sciences.

## 1. Introduction

Earthworms are bilaterally symmetrical, homonomously metameric animals belonging to the phylum Annelida. Within their secondary body cavity (coelom), they contain immune cells known as coelomocytes, which, together with other humoral factors, freely circulate in the coelomic fluid [1]. Several classifications of coelomocytes have been proposed, ranging from early systems in which coelomocytes were named according to mammalian immune cells with analogous functions [2], through classifications distinguishing amoebocytes and eleocytes [3], to more recent classifications suggesting the existence of three or more distinct coelomocyte types [4]. Coelomocytes exhibit numerous characteristics, only some of which are highlighted in this study. They are capable of proliferation, adaptation to different temperatures, regeneration, greenish fluorescence induced by the presence of riboflavin (vitamin B2), opioid-mediated modulation, and survival under conditions of induced microgravity [3,5,6,7,8]. Coelomocytes are also characterised by their dependence on calcium and their sensitivity to the toxic effects of heavy metals and other soil pollutants [9,10]. Some of the principal functions of earthworm coelomocytes include mediation of cellular and humoral immunity, as well as protection against cellular stress. Furthermore, they have been shown to possess antifungal, antimicrobial, and cytolytic activities. Major functions associated with cellular immunity include phagocytosis, formation and extrusion of brown bodies, rejection of foreign tissue, formation of extracellular traps, and cytotoxic activity against mammalian tumour cells [11,12,13,14]. Humoral immunity involves several humoral factors, including lysenin, pattern recognition receptors (Toll-like receptors, lipopolysaccharide-binding proteins, and coelomic cytolytic factor 1), serine proteases, perforin, and cellulase, as well as antimicrobial proteins and peptides [15]. Coelomocytes and coelomic fluid are utilised in various fields of research, including soil quality assessment, as well as in the potential development of compounds such as antifungal agents, antipyretics, anti-inflammatory drugs, and, more recently, biopesticides [16,17,18].

**Figure 1:**
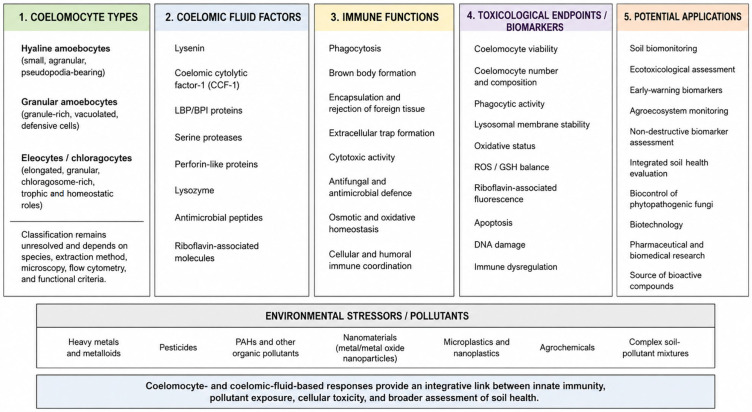
Schematic overview of the relationships among earthworm coelomocyte types, coelomic fluid factors, immune functions, toxicological endpoints/biomarkers, environmental stressors, and potential applications.

Given the increasing interest in earthworms as models in comparative immunology, ecotoxicology, and applied biotechnology, there is a need to integrate the currently fragmented knowledge on coelomocytes and coelomic fluid into a unified framework. Therefore, the aim of this review was to synthesise current evidence on the classification, biological characteristics, immune functions, toxicological responses, and applied potential of earthworm coelomocytes and coelomic fluid. Particular emphasis is placed on their role as sensitive indicators of environmental stress and as sources of bioactive molecules with potential relevance for soil biomonitoring, biocontrol, and biomedical research. By connecting cellular immunity, humoral defence, pollutant sensitivity, and applied research perspectives, this review highlights earthworm coelomocytes as multifunctional biological tools at the interface between organismal health and ecosystem quality. To provide a conceptual overview of the review, Figure 1 summarises the main relationships among coelomocyte types, coelomic fluid factors, immune functions, toxicological endpoints, environmental stressors, and potential research applications.

## 2. Methods and Literature Synthesis Framework

This study was designed as a narrative review aimed at integrating current knowledge on earthworm coelomocytes and coelomic fluid from the perspectives of comparative immunology, ecotoxicology, environmental biomonitoring, and applied biomedical and biotechnological research. The literature search was exploratory, and the literature collection covered all available publication years up to 20 May 2026, which was the date of the final search.

Relevant literature was identified through searches of Web of Science, Scopus, PubMed, and Google Scholar. Search terms included combinations of the following keywords and phrases: “earthworm coelomocytes”, “coelomic fluid”, “earthworm immunity”, “cellular immunity”, “humoral immunity”, “innate immunity”, “lysenin”, “coelomic cytolytic factor”, “earthworm toxicology”, “coelomocyte toxicity”, “earthworm biomarkers”, “soil pollution”, “heavy metals”, “pesticides”, “nanomaterials”, “microplastics”, “antimicrobial peptides”, “antifungal activity”, “cytotoxic activity”, “biocontrol”, and “pharmaceutical application”. Broader searches were also conducted using combinations such as “earthworm* AND coelomocyte*”, “earthworm* AND coelomic fluid”, “coelomocyte* AND pollutant*”, “coelomocyte* AND biomarker*”, and “coelomic fluid AND antimicrobial OR antifungal OR cytotoxic”.

Peer-reviewed original research articles, review articles, and relevant book chapters were considered. Studies were selected for inclusion when they provided information on coelomocyte classification, morphology or physiology, cellular or humoral immune mechanisms, responses to environmental pollutants, biomarker relevance, or potential biomedical, agricultural, or biotechnological applications of coelomocytes or coelomic fluid. Publications were not considered further when they were clearly unrelated to earthworm coelomocytes or coelomic fluid, focused only on general earthworm ecology without immune or toxicological relevance, or did not provide information relevant to the thematic scope of the review. Additional references were identified through citation tracking of relevant publications.

The reviewed literature was organised thematically according to the main conceptual sections of the manuscript: earthworm and coelomic fluid biology; coelomocyte classification; biological characteristics of coelomocytes; immune functions; toxicological responses grouped by pollutant class and cellular endpoint; and applied research fields including soil biomonitoring, biocontrol, and pharmaceutical or biotechnological applications.

## 3. Earthworms

Earthworms are metamerically segmented oligochaetes belonging to the phylum Annelida, class Clitellata, and represent part of the more than 8000 species comprising the subclass Oligochaeta. They are distributed worldwide and inhabit nearly all environments except those characterised by extreme climatic conditions, such as hot and cold deserts [19,20]. Their segmented body organisation is closely associated with the presence of a secondary body cavity, the coelom, which is situated between the body wall musculature and the digestive tract [1]. In earthworms, the coelom is divided into segmentally arranged coelomic sacs lined by mesothelium and separated by septa, with openings that allow the passage of coelomic fluid and dissolved substances between segments [1,21]. This anatomical organisation is directly relevant to coelomocyte biology because coelomocytes circulate freely within the coelomic fluid and interact with both cellular and humoral components of the internal environment. Coelomic fluid also functions as a hydrostatic skeleton, supporting locomotion through coordinated activity of circular and longitudinal muscles while simultaneously providing a medium for immune surveillance, physiological regulation, and responses to environmental stress [1]. In addition to their roles in immunity and homeostasis, coelomocytes and coelomic-fluid-derived molecules have been implicated in regenerative processes. In *Dendrobaena veneta* (Rosa, 1886), experimental evidence indicates that coelomocytes and/or coelomocyte-derived factors contribute to cerebral ganglion regeneration and the subsequent restoration of reproductive function following decerebration [22].

## 4. Coelomocytes and Coelomic Fluid

Coelomocytes are free immune cells present in the earthworm coelomic fluid. These cells participate in the recognition of foreign particles, defence reactions, and phagocytosis [23,24]. Together with their pigmented aggregates, i.e., brown bodies, plasma, and aqueous matrix, they constitute the coelomic fluid [4,25]. All components of the coelomic fluid originate from the mesenchymal tissue surrounding the coelom, i.e., the secondary body cavity [4]. Coelomic fluid is secreted onto the body surface through dorsal pores, i.e., coelomopores, located in the grooves between segments, where, together with the products of mucous glands, it helps maintain moisture and supports physiological functions such as respiration, crawling, burrowing, and protection against predators [1,4,25]. Earthworms also expel coelomic fluid through the same route due to elevated intracoelomic pressure under conditions of physiological stress, which may arise as a consequence of mechanical or chemical irritation [4,25]. Due to the inability to completely close the dorsal pores, pathogens have direct access to the earthworm coelom. It has been reported that coelomic fluid contains approximately 6 × 10^5^ bacteria mL^−1^, while phagocytic cells are more than tenfold more abundant [13]. Together with humoral factors, these cells exhibit antimicrobial activity [26,27]. Humoral factors include lysenin and other lysenin family proteins, such as lysenin-related proteins, including fetidin/lysenin-related protein 2, as well as coelomic cytolytic factor 1 [28,29].

## 5. Classification of Coelomocytes

Despite extensive research, no universally accepted classification system for earthworm coelomocytes has been established. Classification schemes have evolved from early function-based analogies with vertebrate leukocytes to more recent systems based on morphology, ultrastructure, cytochemistry, and flow cytometric characteristics. This diversity of approaches reflects not only biological heterogeneity among coelomocyte populations, but also methodological differences among studies, including the earthworm species examined, extraction procedures used, staining protocols, microscopy resolution, flow cytometric gating strategies, and the functional endpoints selected for analysis. Nevertheless, eleocytes, also referred to as chloragocytes or chloragogenous cells in some classification systems, have remained consistently recognised across most classification systems. Major classification approaches proposed in the literature are summarised in Table 1.

Although terminology and subdivision criteria differ among studies, most classifications recognise amoebocyte-like defensive cells and eleocyte-like trophic cells as principal coelomocyte populations. Advances in microscopy, ultrastructural analyses, and flow cytometry further revealed substantial heterogeneity among amoebocyte populations, particularly regarding granularity, pseudopodia distribution, and cytoplasmic organisation. However, these methodological advances have also made direct comparison among studies more difficult because different techniques do not necessarily identify equivalent cell populations. For example, morphology-based classifications emphasise cell shape, granularity, vacuolisation, and ultrastructure, whereas flow cytometric approaches separate cells according to size, internal complexity, autofluorescence, and staining properties. Functional assays, in contrast, classify or interpret coelomocytes according to activities such as phagocytosis, cytotoxicity, oxidative status, or immune marker expression. Consequently, the same terms, such as amoebocytes, granulocytes, or eleocytes, may not always refer to fully comparable cell populations across studies.

The findings of Manna et al. [35] also expose a broader limitation in the coelomocyte literature: many studies implicitly compare coelomocyte responses across species as if amoebocytes and eleocytes were functionally interchangeable categories. However, differences in habitat, pathogen exposure, soil moisture, organic matter, and pollutant contact may shape baseline coelomocyte composition and activity. Consequently, variation in phagocytosis, autofluorescence, or oxidative status cannot always be interpreted as a pollutant- or treatment-induced effect without considering species-specific immune ecology. This is particularly relevant for ecotoxicological studies, where *Eisenia* models are often generalised to earthworms as a whole. A more robust classification framework would require not only morphological and flow cytometric identification but also functional validation and ecological context.

Recent immunohistochemical evidence further complicates the traditional morphology-based classification of coelomocytes. Alesci et al. [36] demonstrated the presence of TLR2-, CD14-, and α-tubulin-immunoreactive coelomocytes in *L. terrestris*, while incomplete colocalisation of immune markers suggested the existence of functionally distinct coelomocyte subpopulations. Importantly, the study proposed that coelomocyte identity may not be adequately resolved through morphology alone, since cells with similar structural characteristics may differ in immune receptor expression and immunological roles. These findings support the hypothesis that coelomocyte populations represent functionally heterogeneous and potentially plastic immune cell states rather than universally conserved cell categories. The observed expression of phylogenetically conserved immune receptors strengthens the view that several innate immune mechanisms in annelids share evolutionary similarities with vertebrate immune systems.

Methodological variability further complicates comparison among studies. Patil and Biradar [37], comparing coelomic fluid extraction and coelomocyte counts among different epigeic earthworm species, demonstrated that both extraction procedures and species identity may influence the observed coelomocyte composition and relative abundance of cell types. This is important because extraction-induced stress, incomplete recovery of coelomic fluid, differences in body size or coelomic fluid volume, and species-specific sensitivity to handling may all bias the apparent proportion of coelomocyte types. Such variation can affect the interpretation of ecotoxicological studies, particularly when pollutant exposure is evaluated through changes in coelomocyte viability, abundance, fluorescence, phagocytic activity, or oxidative status.

The main unresolved classification problems can therefore be summarised as follows: (I) there is no standardised terminology that can be applied confidently across earthworm species; (II) morphologically similar cells may differ functionally or immunologically; (III) different extraction and analytical methods may alter the observed coelomocyte profile; and (IV) many studies do not combine morphological, molecular, and functional validation. Future work should therefore aim to integrate light and electron microscopy, flow cytometry, immunohistochemical or molecular markers, and functional assays under standardised extraction and handling conditions. Such an integrated approach would improve comparability among studies and strengthen the use of coelomocytes as biomarkers in ecotoxicology and environmental monitoring.

## 6. Characteristics of Coelomocytes

Earthworm coelomocytes display several biologically relevant characteristics, including proliferation and regeneration, seasonal and thermal adaptability, riboflavin-associated fluorescence, opioid-mediated modulation, survival under unusual physical conditions, calcium-dependent signalling, and sensitivity to toxicants. These traits support their role not only in innate immunity but also in environmental adaptation and biomonitoring (Table 2). However, their biomarker relevance differs considerably. Some traits, such as changes in coelomocyte abundance, viability, phagocytic activity, oxidative status, lysosomal stability, and riboflavin-associated fluorescence, are more directly applicable as indicators of pollutant-induced stress. Other characteristics, including seasonal or thermal variability, regeneration after coelomic fluid extraction, and calcium-dependent signalling, are important for understanding immune regulation and stress physiology but require further validation before they can be used as routine biomonitoring endpoints. Opioid-mediated modulation and survival under simulated microgravity should currently be regarded mainly as experimental observations that reveal physiological plasticity rather than established biomarkers.

The findings indicate that coelomocytes are dynamic cells whose abundance, activity, fluorescence, and signalling capacity vary according to physiological state, season, temperature, age, species, and environmental stress. This variability has two important implications. First, several coelomocyte traits are biologically meaningful because they reflect immune activation, cellular homeostasis, or stress adaptation. For example, proliferation and recovery after coelomic fluid extraction are relevant to immune cell renewal and the feasibility of repeated or non-destructive sampling, while calcium signalling reflects cell-type-specific activation pathways involved in immune regulation. Second, the same variability may complicate biomarker interpretation because seasonal cycle, temperature, species identity, age, and extraction method can influence baseline coelomocyte profiles independently of pollutant exposure.

Among the traits discussed, riboflavin-associated fluorescence is one of the most promising biomonitoring endpoints because it can provide a measurable response linked to eleocyte physiology and metal exposure, although it is clearly species-dependent and should not be generalised across all earthworms [8,25,41,42]. Coelomocyte abundance, viability, phagocytic activity, lysosomal stability, and oxidative status also represent robust toxicological endpoints when assessed under standardised conditions and interpreted together with exposure concentration, species, life stage, and environmental context. In contrast, calcium-dependent responses, opioid-mediated modulation, microgravity-associated changes, and riboflavin-associated regeneration are currently better interpreted as mechanistic or experimental observations. These traits improve understanding of coelomocyte physiology and immune regulation but require additional dose–response, interspecies, and field validation before they can be considered reliable biomarkers for environmental monitoring.

### 6.1. Toxicity

(Heavy) metals and metalloids disrupt coelomocyte homeostasis by interfering with intracellular ion balance, oxidative status, membrane integrity, and immune function. Toxic effects include altered coelomocyte viability, impaired phagocytosis, oxidative stress, DNA damage, apoptosis, and disruption of immune signalling pathways. Toxic responses vary depending on metal type, exposure conditions, temperature, and earthworm species (Table 3). However, direct comparison among studies is complicated by major differences in exposure route, concentration, duration, tested species, and endpoint selection. Some studies used isolated coelomocytes or in vitro exposure systems, which are useful for identifying direct cytotoxic, genotoxic, or immunotoxic mechanisms but have limited ecological realism. Other studies used dermal exposure, filter paper contact tests, or polluted soils, which better reflect uptake and organism-level regulation but also introduce additional variability related to soil matrix, metal bioavailability, temperature, exposure duration, and physiological condition of the animals. Therefore, coelomocyte responses should be interpreted in relation to the experimental context.

Among the investigated metals, Cd, Cu, Pb, Hg, and Cr(VI) consistently induced immunotoxic and cytotoxic effects in earthworm coelomocytes, including reduced viability, impaired phagocytosis, oxidative stress, and DNA damage. In contrast, Zn frequently appeared more tightly regulated, although high exposure levels still impaired immune function in some species. Toxic responses were strongly species-dependent and influenced by exposure conditions, temperature, and metal(loid) speciation. For example, in vitro exposure of *L. terrestris* coelomocytes to metal salts for 18 h showed that Hg, Cd, and Zn strongly affected cell viability and phagocytosis, whereas Pb was comparatively better tolerated [30]. In contrast, dermal exposure studies in *E. fetida* and *A. chlorotica* showed that Zn was more efficiently regulated, while Cu, Pb, and Cd reduced coelomocyte numbers and induced stress-related proteins and apoptotic markers [9,45]. These differences illustrate that metal toxicity depends not only on the element itself but also on exposure route, exposure duration, species-specific regulation, and the endpoint measured.

Temperature and exposure matrix further modify coelomocyte responses. In *D. veneta* exposed to polluted soil for 2–4 weeks, stronger adverse effects occurred at 22 °C than at 10 °C, including mortality, reduced free coelomocyte numbers, and increased brown bodies and bacterial load [46]. Similarly, short-term dermal exposure of *D. veneta* showed that metal accumulation and coelomocyte responses depended on both metal identity and temperature, with changes in coelomocyte number, amoebocyte-to-eleocyte ratio, bacterial load, and metallothionein expression [47]. These findings are ecologically important because they show that pollutant effects may interact with environmental temperature and microbial balance.

Chromium and Se studies also demonstrate the importance of endpoint selection. Cr(VI) exposure induced oxidative stress, lysosomal destabilisation, DNA damage, and impaired phagocytosis, indicating that genotoxicity and immune dysfunction can occur together [33,48]. Selenium exposure in *Eisenia andrei* Bouché, 1972, affected oxidative status, efflux pump activity, apoptosis, and expression of stress- and immune-related genes, with responses differing between selenate and selenite forms [49].

### 6.2. Other Pollutants

In addition to heavy metals, earthworm coelomocytes are sensitive to numerous organic pollutants, nanomaterials, and plastic particles. These pollutants impair coelomocyte viability, lysosomal stability, immune activity, oxidative balance, and cellular signalling through diverse mechanisms, including oxidative stress, membrane destabilisation, ion imbalance, and cytotoxic interactions (Table 4). As with metals, however, interpretation depends strongly on whether the study used isolated coelomocytes, whole-animal exposure, freshly polluted or aged soil, single pollutants, or mixtures. These differences affect both toxicological sensitivity and ecological relevance.

These findings demonstrate that coelomocyte responses are not restricted to metal exposure but also occur following contact with pesticides, polycyclic aromatic hydrocarbons, nanomaterials, and plastic particles. Oxidative stress, membrane destabilisation, and impaired immune cell function appear to represent common mechanistic pathways underlying coelomocyte toxicity across chemically diverse pollutants. However, the strength and ecological meaning of these responses differ among studies. Chlorothalonil exposure in *E. fetida* was assessed after 14 days at environmentally relevant soil concentrations, and coelomic fluid appeared to be a particularly sensitive matrix for detecting metabolic perturbation, including changes suggestive of oxidative stress [52]. In contrast, DMBA was tested directly on isolated coelomocytes from *Eisenia hortensis* (Michaelsen, 1890), where it induced ROS production and inhibited phagocytosis, providing mechanistic evidence of oxidative and immunosuppressive effects but under less environmentally complex conditions [50].

Soil ageing and bioavailability are also important. In *E. andrei* exposed to pentachlorophenol-polluted soils, lysosomal membrane stability was reduced across treatments, while coelomocyte subpopulation changes and mortality depended on concentration, ageing period, and exposure duration [53]. This demonstrates that aged or field-like soils may produce toxicological outcomes that cannot be predicted only from nominal concentration. Nanomaterial and mixture studies add another level of complexity. Functional nanocarbon black caused coelomocyte cytotoxicity, while co-exposure with Cd produced antagonistic effects at low doses but synergistic effects at high doses, indicating that combined pollutants may modify intracellular metal availability and toxicity [54]. Finally, microplastic exposure in *E. andrei* linked reduced coelomocyte viability with organism-level effects, including impaired growth and spermatogenesis, as well as biological fragmentation of microplastics into nanoplastics [55]. This type of multilevel response is particularly relevant for soil ecotoxicology because it connects cellular biomarkers with reproductive and ecological consequences.

The toxicological evidence indicates that coelomocytes respond to chemically diverse pollutants through several recurring cellular mechanisms. Metals and metalloids frequently affect ion homeostasis, oxidative balance, phagocytic capacity, viability, and DNA integrity, whereas pesticides, PAHs, nanomaterials, and plastic particles more commonly involve oxidative stress, lysosomal destabilisation, membrane damage, cytotoxicity, and impaired immune cell function. Although the specific responses vary according to species, pollutant type, exposure duration, concentration, and experimental design, several endpoints repeatedly emerge as sensitive indicators of toxicological disturbance: coelomocyte viability, phagocytic activity, lysosomal membrane stability, oxidative status, riboflavin-associated fluorescence, apoptosis, and DNA damage. These endpoints are particularly relevant because they link pollutant exposure with disruption of immune competence and physiological homeostasis, thereby supporting the use of coelomocytes as early-warning biomarkers in soil ecotoxicology. Nevertheless, isolated cellular responses should be interpreted cautiously unless they are supported by organism-level or ecologically relevant endpoints such as survival, growth, reproduction, avoidance behaviour, microbial balance, or tissue-level pathology. Future toxicological studies should therefore place greater emphasis on chronic low-dose exposures, pollutant mixtures, aged or field-polluted soils, multiple earthworm species, and combined cellular, organismal, and ecological endpoints. Such designs would improve the ecological relevance of coelomocyte-based biomarkers and help distinguish general toxicological responses from species- or method-specific effects.

## 7. Functions of Coelomocytes

Coelomocytes and coelomic fluid primarily play a role in immune reactions. Earthworms possess only innate immunity; acquired immunity is absent, and, therefore, immunological memory is also lacking [56]. The components of the earthworm immune system consist of cellular and humoral elements located in the coelomic fluid [15]. The activity of these components is directed towards avoidance of osmotic stress, while more recent studies have shown that they also possess antifungal and antimicrobial activity.

### 7.1. Avoidance of Osmotic Stress

Earthworms are frequently exposed to changes in environmental osmotic parameters due to fluctuating wet–dry conditions in the soil they inhabit. Owing to its low ion concentration, coelomic fluid has the capacity to adapt to osmotic stress. However, Kasschau et al. [57] demonstrated that *E. fetida* coelomocytes exposed to extreme osmolarities for several hours show reduced viability. Osmotic stress affects coelomocytes by changing their shape from round to different filopodial forms, characterised by thin membrane protrusions that increase cell diameter. Microtubules are responsible for enabling the formation of coelomocyte filopodia. Earthworm coelomocytes form four types of filopodia and one type of cytoneme-like podia with one to two strands. Cytonemes are important for communication, aggregation, and transport between cells. Filopodia may therefore help earthworms locate osmotically more favourable environments [57].

### 7.2. Antimicrobial Activity

Earthworm coelomocytes and coelomic fluid participate in antimicrobial defence against fungi and bacteria through both cellular responses and soluble humoral factors. Several studies have demonstrated antifungal activity of earthworm-derived preparations or coelomic fluid. Ansari and Sitaram [58] showed that *E. fetida* exhibits antifungal activity against *Candida albicans*. The coelomic fluid of *E. fetida* and *D. veneta* slows the growth of the phytopathogenic fungus *Fusarium oxysporum* through contact interaction, with *D. veneta* showing a more pronounced, although only slightly different, effect than *E. fetida* [24]. *D. veneta* coelomic fluid also exhibits antifungal activity by damaging the cell wall and inducing apoptotic death in *C. albicans* and *Candida krusei* [59]. In addition, the coelomic fluid of *Eudrilus eugeniae* (Kinberg, 1867) inhibits hyphal growth of several phytopathogenic fungi, including *Rhizoctonia solani*, *F. oxysporum*, *Aspergillus flavus*, and *Verticillium dahliae* [60]. In addition to antifungal activity, earthworm coelomic fluid contains several antimicrobial factors with antibacterial or broader immune-related functions. These include lysozyme, which hydrolyses bacterial cell wall peptidoglycan; antimicrobial peptides such as lumbricin I, PP-1, and related peptides; lysenin family proteins, including lysenin-related proteins such as fetidin/lysenin-related protein 2; coelomic cytolytic factor 1; LBP/BPI proteins involved in recognition of Gram-negative bacterial lipopolysaccharides; and serine proteases and perforin-like molecules involved in cytolytic or immune effector responses [13,15,26,27,59,61,62,63]. These components indicate that earthworm antimicrobial defence is not restricted to direct antifungal activity but involves a broader network of coelomocyte-mediated cellular responses and soluble coelomic fluid factors. However, the precise mechanisms and relative contribution of individual molecules remain incompletely resolved, particularly because many studies use crude coelomic fluid or extracts rather than purified active compounds [24,58,59,60].

### 7.3. Cellular Immunity

Earthworm cellular immunity is primarily mediated by hyaline and granular amoebocytes, which perform complementary defensive functions. Granular amoebocytes participate in encapsulation and natural killer cell-like cytotoxic activity, whereas hyaline amoebocytes mainly exhibit phagocytic activity [22,23,29,50]. In addition to direct elimination of foreign particles and abnormal cells, coelomocytes participate in brown body formation, rejection of foreign tissues, formation of extracellular traps, and cytotoxic responses against tumour cells. The principal mechanisms of earthworm cellular immunity are summarised in Table 5.

#### 7.3.1. Phagocytosis

Phagocytosis represents one of the principal cellular defence mechanisms in earthworms and involves engulfment and degradation of foreign particles by coelomocytes, particularly amoebocytes [1]. Xenogeneic cells are phagocytosed more rapidly than allogeneic cells, suggesting selective recognition of foreign material. Humoral opsonins present in coelomic fluid may enhance phagocytic activity, as pre-incubation with coelomic fluid or mammalian opsonins such as IgG and C3b increases phagocytosis of yeast and synthetic particles [13]. Phagocytosis may also act together with encapsulation during brown body formation.

#### 7.3.2. Formation of Brown Bodies

Brown bodies are multicellular aggregates formed around foreign particles or altered host cells too large to be eliminated by phagocytosis alone. Their formation involves aggregation of amoebocytes and chloragocytes around pathogens, tissue debris, or other foreign material within the coelomic cavity [11]. Granular amoebocytes mainly contribute to encapsulation, whereas hyaline amoebocytes contribute through phagocytic activity. Brown bodies contain melanin and lipofuscin, which participate in pathogen elimination and degradation of altered particles [23,25]. Melanin production occurs through the phenoloxidase cascade, and mature brown bodies may ultimately be removed through autotomy of posterior segments [64].

#### 7.3.3. Rejection of Foreign Tissue

Earthworms are capable of distinguishing between autografts, allografts, and xenografts. Rejection responses involve accumulation of coelomocytes around the graft, followed by encapsulation and cytotoxic degradation of recognised foreign tissue [13]. Xenografts induce stronger responses than autografts and require both cellular and humoral components for complete destruction [65]. Chemotactic attraction of coelomocytes towards tissues of related species has also been demonstrated, suggesting selective recognition mechanisms in earthworm immunity [66].

#### 7.3.4. Extracellular Traps

Extracellular traps (ETs) are extracellular DNA–protein networks composed mainly of chromatin fibres, histone H3, and heat shock proteins [14]. ET formation is induced by several microbial and chemical stimuli, including lipopolysaccharides and zymosan. Formation of ETs involves reactive oxygen species production, chromatin decondensation, histone citrullination, and extrusion of DNA together with granular components into the extracellular space. ETs contribute to immobilisation and elimination of pathogens, similarly to neutrophil extracellular traps in vertebrates [14]. Recent studies further demonstrate that earthworm coelomocyte responses involve highly coordinated interactions between cellular and humoral immune mechanisms. Topa et al. [67] showed that ETs released by *E. andrei* coelomocytes actively participate in encapsulation processes and brown body formation following exposure to LPS-coated particles. The study demonstrated that ET formation was associated with increased expression of pattern recognition molecules such as CCF-1 and LBP/BPI, while protease activity and humoral coelomic fluid components were required for efficient encapsulation and ET release. Interestingly, ET formation occurred largely independently of NADPH oxidase activity, suggesting that earthworm coelomocytes may utilise both ROS-dependent and ROS-independent immune pathways analogous to those described in vertebrate innate immunity.

#### 7.3.5. Killing of Tumour Cells

Earthworm coelomocytes exhibit cytotoxic activity against tumour and foreign target cells, including K562, HeLa, Hep-2, PC-12, and PA317 cell lines [13,16]. Small granular coelomocytes participate in direct cytotoxic interactions, whereas large agranular phagocytic coelomocytes remove cellular debris following target cell destruction. This activity resembles natural killer cell-like responses in vertebrates and involves cytotoxic effector molecules and contact-mediated killing mechanisms [12,44]. Exposure to immunotoxic compounds such as 7,12-dimethylbenz[*a*]anthracene reduces this cytotoxic activity [12].

### 7.4. Humoral Immunity

Humoral immunity in earthworms is mediated by biologically active macromolecules present in the coelomic fluid. These humoral factors are primarily produced by chloragocytes, although granular coelomocytes may also contribute to their synthesis [23]. Earthworm humoral factors exhibit antimicrobial, haemolytic, cytolytic, antioxidant, and immunomodulatory activities and participate in pathogen recognition, coagulation, melanisation, and direct cytotoxic defence [13,15].

#### Lysenin

Lysenin is one of the best-characterised humoral immune factors in earthworms and is predominantly synthesised during maturation of chloragocytes into free coelomocytes [68]. It exhibits haemolytic and cytotoxic activity against insect haemocytes, vertebrate fibroblasts, and tumour cells [15,69]. Lysenin specifically binds sphingomyelin, a phospholipid predominantly located on vertebrate cell membranes, and subsequently forms oligomeric pores that induce cell lysis [61,68]. Earthworms themselves lack sphingomyelin, which may represent a protective adaptation preventing autolysis by lysenin [15]. Hayashi et al. demonstrated that *E. fetida* coelomic proteins form a species-specific biomolecular corona around silver nanoparticles, and that lysenin contributes critically to coelomocyte–nanoparticle interactions and nanoparticle accumulation [70]. Lysenin expression is influenced by bacterial exposure, with Gram-positive bacteria inducing stronger expression than Gram-negative bacteria [28]. The principal humoral immune factors identified in earthworms are summarised in Table 6.

## 8. Application in Research

In research, earthworms and their coelomic fluid are most commonly used to assess soil pollution because of their constant contact with soil and the simplicity of sampling. In addition to studies related to soil pollution, further application in other research fields has also been explored, including the potential use of earthworm coelomic fluid and coelomocyte-derived molecules in the biocontrol of phytopathogenic fungi. Earthworm coelomocytes may also be used in pharmaceutical research because they contain active substances with antifungal, cytotoxic, anti-inflammatory, or antipyretic activity.

### 8.1. Soil Pollution and Integrated Biomonitoring

Gautam et al. [74] state that pollution of soil inhabited by earthworms results in immunocompromise and suppression of innate immunity, specifically reduced phagocytic and lysozyme activity, degradation of lysosomal membranes, increased phosphatase activity, premature autolysis, and increased coelomocyte necrosis. Earthworms with a high number of chloragocytes or eleocytes, rich in riboflavin, are a suitable choice for monitoring metal pollution in soil using fluorescence. Metal pollution of soil affects earthworm immunity and, consequently, cell viability. A reduction in the number of viable eleocytes is accompanied by reduced fluorescence intensity. *A. chlorotica* shows high sensitivity to metal exposure, expressed specifically through changes in eleocyte fluorescence. This species is a suitable organism for monitoring metal pollution in soil not only because of its sensitivity but also because of its wide distribution [19]. High concentrations of Cd, Cr, Pb, and Hg have been recorded in tropical earthworm species such as *Metaphire posthuma* (Vaillant, 1868) [74]. Earthworm species suitable as bioindicators of soil pollution include *D. veneta*, *L. terrestris*, *E. fetida*, *E. andrei*, and *E. hortensis*. These species show sensitivity to Pb, Hg, Zn, Cu, Cd, Cr, Se, chlorothalonil, pentachlorophenol, 7,12-dimethylbenz[*a*]anthracene, functional nanocarbon black, and microplastics and nanoplastics.

However, earthworms should not be viewed only as sources of isolated cellular biomarkers but also as sentinel organisms that integrate pollutant exposure across cellular, tissue, organismal, and ecological levels. Coelomocyte-based endpoints such as viability, phagocytic activity, lysosomal membrane stability, oxidative status, micronucleus frequency, morphometric alterations, cytoskeletal changes, and riboflavin-associated fluorescence provide early information on immune competence and cellular homeostasis. Their interpretation becomes stronger when these responses are linked with tissue-level alterations, such as epithelial or muscular damage, inflammatory responses, fibrosis, mucin secretion, or histopathological lesions, and with organism-level endpoints, such as survival, growth, biomass change, reproduction, avoidance behaviour, and overall physiological condition.

Recent multilevel approaches illustrate the value of this integrated perspective. Power et al. [75] assessed the effects of conventional polyethylene microplastics and biodegradable and compostable microplastics on *E. fetida* by combining classical ecotoxicological endpoints with an ecopathological approach. Although abiotic soil properties were only weakly affected, exposed earthworms showed increased mortality, reduced biomass, changes in offspring production, and tissue-level alterations, including epidermal and muscular damage, inflammatory or degenerative phenomena, collagen deposition, and altered mucin secretion. Such findings demonstrate that histopathological and ecopathological endpoints can reveal sublethal tissue and cellular damage that helps explain organism-level effects and may anticipate broader impairment of earthworm function in soil ecosystems. Similarly, Calisi et al. [76] demonstrated the usefulness of coelomic fluid as a source of non-destructive biomarkers in *L. terrestris* exposed to copper sulfate and chlorpyrifos. The authors measured metallothionein concentration, acetylcholinesterase activity, lysosomal membrane stability, micronucleus frequency, granulocyte morphometric alterations, and cytoskeleton polymerisation, showing that coelomic fluid-based endpoints can detect significant responses to agrochemicals without sacrificing the animals. This approach is particularly relevant for agroecosystem monitoring because it allows repeated or less invasive assessment of non-target sentinel species and connects cellular immune responses with organism health and soil pollution.

Therefore, coelomocyte and coelomic fluid biomarkers are most informative when included in integrated biomonitoring frameworks rather than interpreted as isolated endpoints. A combined assessment of cellular biomarkers, tissue pathology, organism-level performance, and ecological functions, such as organic matter decomposition, nutrient cycling, soil aeration, and microbial regulation, would provide a more complete evaluation of soil health. In this context, earthworms represent valuable sentinel organisms because their coelomic fluid and coelomocytes respond rapidly to pollutants, while their growth, reproduction, behaviour, and tissue integrity reflect broader ecotoxicological and ecopathological consequences.

### 8.2. Potential Application in the Biocontrol of Phytopathogenic Fungi

Soil is a complex habitat in which interactions among earthworms, phytopathogenic fungi, and plants may influence plant health and productivity. Van Groenigen et al. [77] state that earthworms positively affect plant growth in five ways: by modifying soil structure, increasing nutrient availability, producing growth regulators, stimulating symbioses, and controlling pests and diseases. Phytopathogenic fungi are fungi that, through interaction with plants, destroy their cell walls. Earthworms, or more precisely, their coelomic fluid, have been shown to exhibit antifungal activity and slow the growth of phytopathogenic fungi, thereby positively affecting plant growth [77,78]. The coelomic fluid of *E. fetida*, *D. veneta*, and *E. eugeniae* slows the growth of several phytopathogenic fungi through contact interaction, including *R. solani*, *F. oxysporum*, *A. flavus*, and *V. dahliae* [24,60]. However, these findings should be interpreted primarily as evidence of antifungal activity under controlled experimental conditions rather than as direct proof of practical agricultural applicability. Although the antifungal activity of coelomic fluid and coelomocytes has been demonstrated, Fiołka et al. [59] note that the exact mechanism of this activity remains unknown. Moreover, the development of earthworm-derived compounds as natural fungicidal or biocontrol agents would require several additional steps, including identification and purification of the active molecules, assessment of their stability under storage and field conditions, development of suitable formulations, and optimisation of delivery methods in soil or on plant surfaces. Specificity toward target phytopathogens would also need to be evaluated, together with potential effects on beneficial soil microorganisms, plants, earthworms themselves, and other non-target organisms. Field validation under realistic agricultural conditions is especially important because soil physicochemical properties, microbial communities, organic matter, moisture, temperature, and degradation processes may strongly influence the persistence and efficacy of coelomic fluid-derived compounds. Earthworms, together with their coelomic fluid, therefore offer potential as sources of bioactive antifungal compounds but should not yet be regarded as ready-to-use agricultural fungicides. Their possible contribution to environmentally oriented biocontrol is relevant, but practical application will require that future studies address the mechanism of action, formulation, environmental safety, delivery, persistence and efficacy under field conditions.

### 8.3. Pharmaceutical Application

Earthworm coelomic fluid and coelomocyte-derived molecules have attracted attention because of their reported antimicrobial, antibiofilm, cytotoxic, anti-inflammatory, antipyretic, cardiovascular, and cosmeceutical activities. However, most of the available evidence is based on in vitro assays, cell culture systems, or preliminary experimental models. Therefore, these effects should be interpreted as preliminary bioactivity or preclinical potential rather than as evidence of established therapeutic, clinical, or cosmetic applicability.

#### 8.3.1. Antimicrobial and Antibiofilm Activity

In humans, fungi may cause infections of the skin, mucosa, or systemic infections. Ansari and Sitaram [58] demonstrated the antifungal activity of earthworms by exposing *C. albicans* to a preparation made from *E. fetida* individuals. In addition to *E. fetida*, fungicidal activity against *C. albicans* and *C. krusei* has also been shown by *D. veneta*, *P. corethrurus*, and *M. houletti* [59]. Based on these findings, earthworm-derived preparations may represent a potential source of antimicrobial compounds, but their use in the treatment of human candidiasis remains speculative and would require further pharmacological, toxicological, and clinical validation. Recent studies further suggest that earthworm-derived bioactive compounds may interfere not only with microbial growth but also with biofilm formation. Hussain et al. [79] demonstrated that coelomic fluid and body paste from *M. posthuma* inhibited biofilm formation in several pathogenic bacterial species, including *Pseudomonas aeruginosa*, *Escherichia coli*, *Staphylococcus aureus*, and *Klebsiella pneumoniae*, in a concentration-dependent manner. However, the study was limited to in vitro assays, while the specific bioactive compounds and molecular mechanisms responsible for antibiofilm activity remain largely unresolved. Moreover, substantial methodological variability among antibiofilm studies currently limits direct comparison of efficacy across different earthworm species, extracts, and experimental systems. Before antimicrobial or antibiofilm applications can be considered translationally relevant, future studies should identify the active molecules, standardise extraction and purification procedures, determine dose–response relationships, evaluate stability and bioavailability, and assess safety, immunogenicity, and selectivity toward microbial rather than host cells.

#### 8.3.2. Cytotoxic Activity

Coelomic fluid contains cytolytic and haemagglutinating molecules, which are produced, stored, and released by coelomocytes. Coelomocyte lysates contain proteins or peptides with cytotoxic activity, which can be reduced by heating and digestion with proteinase K and inactivated by potassium chloride (KCl). Coelomocytes and coelomic fluid use cytolytic molecules in the same cell death mechanism. Cytotoxic proteins remove target eukaryotic cells through rapid reactions. Cell lines such as HeLa, Hep-2, PC-12, and PA317 exposed to coelomic fluid and coelomocytes show necrosis-like damage. Irreversible cell damage begins with nuclear swelling and continues with granule formation in the cytoplasm; finally, entire cells become deformed. The antitumour and cytotoxic activities of coelomocytes and the factors they produce appear promising for research into potential drug development [16]. Moreover, Haque et al. [80] summarised numerous in vitro and limited in vivo studies demonstrating antiproliferative, proapoptotic, and cytotoxic effects of coelomic fluid-derived compounds against different cancer cell lines. However, the authors also highlighted several important limitations, including the predominance of in vitro models, relatively high effective concentrations compared to conventional chemotherapeutics, incomplete mechanistic understanding, and limited standardisation of extraction and purification procedures [80]. Additional limitations include incomplete molecular characterisation of active fractions, uncertain selectivity toward cancer versus normal cells, limited information on pharmacokinetics and bioavailability, and insufficient validation of reproducible dose–response effects. Beyond the aforementioned properties, recent studies suggest that coelomic fluid-derived compounds may also possess cardiovascular bioactivity. Poniedziałek et al. [81] demonstrated that a polysaccharide–protein complex isolated from *D. veneta* coelomic fluid inhibited platelet aggregation through multiple pathways, including P2Y12, COX-1, and PAR-1 signalling, without inducing apparent coagulopathy or cytotoxicity under in vitro conditions. Nevertheless, the authors emphasised that the findings remain limited to experimental in vitro models, while the bioavailability, active molecular components, long-term safety, and translational relevance of these compounds remain unresolved. Consequently, despite promising preliminary findings, the translational and clinical relevance of earthworm-derived anticancer or cardiovascular-active compounds remains uncertain and requires substantially more rigorous pharmacological and toxicological validation.

#### 8.3.3. Anti-Inflammatory and Antipyretic Activity

In traditional medicine, earthworms have been used for centuries to treat fever, abdominal and neck pain, and neurological and digestive disorders. The earthworm *Lampito mauritii* Kinberg, 1867 exhibits a range of scientifically demonstrated effects in mammalian organisms; for example, in the form of a paste, it possesses anti-ulcer and antioxidant properties. An extract of *L. mauritii* suppresses the formation of oedema induced by histamine, a mediator of inflammatory reactions, thereby confirming its anti-inflammatory activity. The antipyretic property of earthworm extract has been observed as a reduction in fungus-induced fever [17]. Although these findings support the presence of biologically active compounds, the evidence remains preliminary. Further studies are required to identify the responsible molecules, clarify mechanisms of action, establish dose–response relationships, evaluate toxicity and immunogenicity, and determine whether these effects are reproducible in standardised preclinical models. Therefore, anti-inflammatory and antipyretic effects should currently be regarded as experimental bioactivities instead of validated therapeutic applications.

#### 8.3.4. Active Substances

Enzymes, active proteins, and peptides have been identified in earthworms. Active enzymes include fibrinolytic enzymes, which degrade fibrin and activate plasminogen; intestinal digestive enzymes, such as oligosaccharidases and heterosidases, which degrade heterosides, including N-acetylglucosamine, oligosaccharides, such as maltose and laminaribiose, and polysaccharides; glycolytic enzymes involved in digestion and originating from digested microflora; phosphatases, including phosphomonoesterases and phosphodiesterases, which hydrolyse *p*-nitrophenyl phosphate; cellulases, which degrade cellulose in the intestine; and acetylesterases. Active proteins and peptides include haemolytic antibacterial proteins, such as fetidins and coelomic cytolytic factor 1; antibacterial proteins without haemolytic characteristics, including lumbricin 1, PP-1, and antibacterial tetradecapeptide; antibacterial peptides, such as F-1, F-2, and ECP5-1; lysozyme, which hydrolyses mucopolysaccharides of bacterial cell walls; earthworm metallothionein; myosin; cytochrome; antigen-binding proteins; and agglutinins and haemolysins [18]. Owing to the increasing demand for drugs, earthworms, which have been used in traditional medicine for centuries, represent a promising source of bioactive substances with potential pharmaceutical application. However, the presence of bioactive molecules alone does not establish translational applicability. For most earthworm-derived compounds, major gaps remain regarding molecular characterisation, purity, extraction reproducibility, stability, bioavailability, pharmacokinetics, safety, immunogenicity, and validated dose–response relationships.

Recent studies have also explored potential cosmeceutical applications of earthworm coelomic fluid. Imeni et al. [82] developed a serum formulation containing coelomic fluid from *E. fetida*/*E. andrei* and reported antibacterial, antioxidant, and fibroblast-restorative effects together with low cytotoxicity under in vitro conditions. However, the proposed anti-ageing and regenerative effects remain based primarily on cell culture assays and preliminary formulation testing, while the active molecular components, long-term safety, skin permeability, immunogenicity, and clinical efficacy of such preparations remain largely unresolved. These limitations highlight the substantial gap that still exists between experimental bioactivity studies and clinically validated therapeutic or cosmetic applications. Overall, earthworm-derived molecules should currently be considered promising candidates for further biochemical and preclinical investigation, rather than established antimicrobial, anticancer, cardiovascular, anti-inflammatory, antipyretic, or cosmeceutical agents.

## 9. Conclusions and Future Directions

Earthworm coelomocytes and coelomic fluid represent a highly specialised and evolutionarily conserved defence system that integrates cellular and humoral immune mechanisms with remarkable physiological plasticity. Although earthworms lack adaptive immunity, the diversity of coelomocyte populations and the broad spectrum of biologically active molecules present in coelomic fluid enable efficient recognition and elimination of pathogens, regulation of oxidative and osmotic stress, tissue repair, and maintenance of organismal homeostasis. The evidence summarised in this review demonstrates that coelomocytes are not only key mediators of innate immunity but also highly sensitive indicators of environmental disturbance.

Numerous studies have shown that exposure to heavy metals, pesticides, nanomaterials, microplastics, and other pollutants alters coelomocyte viability, phagocytic activity, oxidative status, lysosomal stability, fluorescence, and expression of immune-related molecules. These responses occur at cellular and molecular levels before visible effects appear at the organismal level, confirming the value of earthworm coelomocytes as early-warning biomarkers of soil pollution. Their sensitivity, accessibility, and functional diversity make earthworms particularly suitable for ecotoxicological research and environmental biomonitoring, especially in terrestrial ecosystems increasingly exposed to complex pollutant mixtures. At the same time, the biological properties of coelomocytes and coelomic fluid extend far beyond environmental monitoring. Molecules such as lysenin, coelomic cytolytic factor 1, perforin-like proteins, serine proteases, lysozyme, and antimicrobial peptides exhibit antifungal, antimicrobial, cytotoxic, anti-inflammatory, and immunomodulatory activities, although most applied evidence remains preliminary and requires further biochemical, toxicological, and translational validation. Therefore, earthworm-derived compounds should currently be regarded as promising sources of bioactive molecules rather than established biopesticides, pharmaceutical, or cosmetic agents. Despite significant progress, gaps in knowledge remain. The lack of a universally accepted coelomocyte classification system, limited understanding of signalling pathways and intercellular communication, and insufficient integration of molecular, physiological, and ecological approaches continue to constrain interpretation of immune and toxicological responses. Future studies should prioritise several concrete directions: (I) coelomocyte classification should be standardised by combining morphology, ultrastructure, flow cytometry, molecular or immunohistochemical markers, and functional assays; (II) extraction, handling, counting, and viability assessment methods should be harmonised to improve comparability among studies and reduce methodological bias; (III) coelomocyte-based biomarker panels should be validated across species, pollutant classes, exposure durations, and soil types, rather than relying on single endpoints in isolated experimental systems; (IV) cellular endpoints such as viability, lysosomal stability, phagocytosis, oxidative status, riboflavin-associated fluorescence, apoptosis, and DNA damage should be linked more explicitly with tissue-level, organism-level, and ecological responses, including histopathology, growth, reproduction, behaviour, survival, and soil function indicators; (V) omics approaches, including transcriptomics, proteomics, metabolomics, and microbiome analyses, should be integrated with classical biomarker endpoints to clarify mechanisms of coelomocyte activation, immune disruption, and pollutant-induced stress; (VI) future ecotoxicological studies should increasingly test environmentally realistic pollutant mixtures, including metals, pesticides, microplastics, nanomaterials, and organic pollutants, under exposure scenarios that reflect actual soil conditions, ageing processes, bioavailability, and long-term ecological risk.

An additional limitation is that much of the available experimental evidence is derived from a relatively small number of model or laboratory-maintained species, particularly *E. fetida* and *E. andrei*. Although these species are highly useful because they are easy to culture, widely used in ecotoxicological testing, and responsive to pollutant exposure, they do not represent the full ecological, physiological, and immunological diversity of earthworms. Extrapolating findings from *Eisenia* spp. to other ecological groups, such as epigeic, endogeic, and anecic earthworms, should therefore be done cautiously. Species may differ in habitat, feeding strategy, coelomic fluid volume, baseline coelomocyte composition, fluorescence intensity, immune responsiveness, pollutant exposure routes, and tolerance mechanisms. Broader comparative studies across earthworm taxa and ecological groups are needed to determine which coelomocyte-based endpoints are generalisable and which are species-specific.

Overall, earthworm coelomocytes and coelomic fluid should be regarded not only as components of annelid immunity but also as valuable model systems linking immunology, ecotoxicology, environmental monitoring, and applied biomedical sciences. Their multifunctional nature and high responsiveness to environmental change position them as increasingly important tools for understanding ecosystem health provided that future research moves toward standardised methods, multilevel biomarker validation, mechanistic interpretation, and environmentally realistic testing.

## Figures and Tables

**Table 1:** Major classification approaches proposed for earthworm coelomocytes, including morphological, ultrastructural, cytochemical, and functional characteristics described in different studies.

Coelomocyte Types	Main Characteristics	Proposed Functions/Notes	Reference
Basophils, neutrophils, acidophils, granulocytes, chloragogenous cells	Functional analogy with vertebrate leukocytes	Early functional classification system	[2]
Eleocytes and amoebocytes	Eleocytes: elongated, granular, morula-shaped (~23 μm); amoebocytes: round/oval, adherent, pseudopodia-bearing (5–15 μm)	Amoebocytes suggested to have more prominent defensive role	[11,25,30]
Hyaline and granular amoebocytes	Granular amoebocytes contain translucent granules; hyaline amoebocytes possess pseudopodia, vacuoles, Golgi apparatus, agranular cytoplasm	Subdivision based on ultrastructure and cytochemistry	[3,23,28]
Three or four coelomocyte types	Distinguished using flow cytometry and light microscopy based on size, granularity, and staining	Demonstrated coelomocyte heterogeneity in *E. fetida*	[30,31]
Eleocytes, amoebocytes, granulocytes	Detailed ultrastructural classification; differences in pseudopodia, granules, vacuoles, and organelles	Morphology- and behaviour-based classification	[32,33]
Agranular, large agranular, granular, vacuolated coelomocytes	Differences in chromatin distribution, vacuoles, granules, and cytoplasmic organisation	One of the earliest ultrastructural classifications	[34]
Amoebocytes, mucocytes, circular cells, chloragogenous cells	Amoebocytes spherical and phagocytic; mucocytes elongated; circular cells poorly characterised; chloragocytes rich in chloragosomes	Modern morphology- and function-based classification	[4]
Hyaline amoebocytes, granular amoebocytes, eleocytes	Distinguished using flow cytometry, morphometry, autofluorescence, oxidative and phagocytic characteristics across ecologically distinct earthworm species	Demonstrated species- and habitat-associated functional heterogeneity in coelomocytes	[35]

**Table 2:** Principal biological and physiological characteristics of earthworm coelomocytes, including proliferation, regeneration, fluorescence, calcium signalling, environmental adaptation, and responses to stress.

Characteristic	Key Observations	Biological Significance	Species/Model	Ref.
Proliferation/origin	Coelomocytes originate from cells lining the coelom; free coelomocyte proliferation remains unclear. Antigenic stimulation changes the number of free coelomocytes, phagocytic cells, and antigen-binding cells.	Suggests recruitment and differentiation of precursor cells during immune activation.	Earthworms; *L. terrestris*	[3,6]
Seasonal proliferation/maturation	Granular amoebocytes increase in spring and decrease in winter.	Indicates possible endogenous seasonal cycle of coelomocyte proliferation or maturation.	*L. terrestris*	[3]
Thermal adaptability	Winter coelomocytes perform better at lower temperatures, while summer coelomocytes perform better at higher temperatures. Acclimatisation affects membrane fluidity and phospholipid fatty acid composition.	Important for immune defence in ectothermic organisms exposed to seasonal temperature variation.	*L. terrestris*, *A. chlorotica*	[3,38,39]
Regeneration after coelomic fluid extraction	Coelomocyte numbers return to control levels after 2–4 weeks at room temperature but not after 6 weeks at lower temperatures. Juveniles recover more efficiently than adults.	Demonstrates temperature- and age-dependent immune cell restoration.	Adult and juvenile earthworms	[5,40]
Riboflavin-associated regeneration	Riboflavin may stimulate regeneration; coelomocyte-derived molecules may support regeneration of the subpharyngeal ganglion.	Links coelomocytes with broader tissue and nervous system regeneration.	*E. eugeniae*; earthworms	[22,40]
Riboflavin-induced fluorescence	Riboflavin, FMN, and FAD produce green fluorescence. Autofluorescence occurs mainly in eleocytes/chloragogen-derived coelomocytes.	Useful marker of eleocyte physiology and potential biomonitoring endpoint.	*A. chlorotica*, *D. rubidus*, *E. fetida*, *Octolasion* spp.	[8,25,41]
Species differences in fluorescence	Some species show weak or absent autofluorescence. Species with more coelomocytes relative to body mass often show stronger autofluorescence.	Indicates that fluorescence is species-dependent and should not be generalised across earthworms.	*L. castaneus*, *L. festivus*, *L. rubellus*, *L. terrestris*, *A. caliginosa*, *A. longa*	[25]
Effects of metals on riboflavin fluorescence	FeCl_3_ does not reduce riboflavin content in *E. fetida*. Zn/Pb and Ni/Cu pollution decreases riboflavin content in *D. rubidus*, with effects persisting after transfer to unpolluted soil.	Supports the use of riboflavin fluorescence as a sensitive but context-dependent pollution response marker.	*E. fetida*, *D. rubidus*	[8,41,42]
Opioid-mediated modulation	Opioids modulate amoebocyte activity. Met-enkephalin enhances phagocytosis, aggregation, granulation, and conformational changes; naloxone inhibits these effects.	Suggests neuroimmune-like regulation and possible opioid receptors on amoebocytes.	Earthworm amoebocytes	[7]
Survival under microgravity	Simulated microgravity increased coelomocyte number and granule/mitochondria-like structures after 20–44 h, without altering phagocytic, haemolytic, or protease activity.	Shows resilience under unusual physical stress, although mechanisms remain unresolved.	*E. fetida*	[43]
Calcium dependence	Calcium acts as a second messenger. Eleocytes contain high Ca levels; amoebocytes show moderate levels. Calreticulin was identified in *E. fetida* tissues and coelomocytes.	Indicates a role for calcium signalling in immune cell regulation.	*E. fetida*	[10,44]
Calcium signalling responses	Ionomycin and thapsigargin increase intracellular Ca^2+^ in amoebocytes. Phytohaemagglutinin induces Ca^2+^ signalling, while concanavalin A and LPS may stimulate proliferation through Ca^2+^-independent pathways. Eleocytes show no comparable response.	Suggests cell-type-specific calcium mobilisation and possible functional differences between amoebocytes and eleocytes.	*E. fetida*, *A. caliginosa*	[10]

**Table 3:** Effects of heavy metals and metalloids on earthworm coelomocytes, including alterations in viability, oxidative status, immune function, phagocytosis, and cellular homeostasis.

Stressor	Species	Exposure Design	Main Endpoints	Key Coelomocyte Effects and Interpretation	Ref.
Hg, methyl-Hg, Cd, Zn, Pb	*L. terrestris*	Isolated coelomocytes; in vitro; 10^−4^–10^−9^ M; 18 h; 15 °C	Viability; phagocytosis; flow cytometry	Hg, Cd, and Zn reduced viability and phagocytosis; Pb was better tolerated. Large coelomocytes were particularly sensitive to Hg, showing metal-specific immunotoxicity.	[30]
Cu, Pb, Cd, Zn	*A. chlorotica*, *E. fetida*	Dermal exposure to metal ions	Metal accumulation; coelomocyte number; stress proteins; apoptosis markers	Cu, Pb, and Cd reduced coelomocyte number; Zn was more efficiently regulated. Responses differed between species, with *A. chlorotica* more sensitive to Cu.	[45]
Zn, Cu, Pb, Cd	*E. fetida*	Filter paper dermal exposure; 3 days	Coelomocyte number; HSP70/HSP72; metallothionein	Cu, Pb, and Cd decreased coelomocyte number and induced stress protein responses, supporting the biomarker relevance of metallothionein and heat shock proteins.	[9]
Zn, Pb, Cd	*D. veneta*	Polluted soil exposure; 2–4 weeks; 10 or 22 °C	Coelomocyte number; brown bodies; bacterial load; mortality	Strongest effects occurred after 4 weeks at 22 °C in heavily polluted soil, with reduced free coelomocytes, increased brown bodies and bacteria, and 30% mortality.	[46]
Zn, Cu, Cd	*D. veneta*	Dermal exposure; 3 days; 10 or 22 °C	Coelomocyte number; amoebocyte/eleocyte ratio; bacterial load; metallothionein	Zn did not reduce coelomocyte number, whereas Cd and Cu did. Responses depended on metal identity and temperature, indicating altered immunocompetence/pathogen balance.	[47]
Cr(VI)	*D. curgensis*	In vitro and in vivo Cr(VI) exposure; sublethal concentrations	Genotoxicity; comet assay	Cr(VI) induced DNA damage, with dose-dependent effects in vitro and non-linear responses in vivo, possibly due to DNA cross-linking at higher concentrations.	[33]
Cr(VI)	*E. andrei*	Paper contact exposure; 2, 15, 30 µg mL^−1^; 1 and 3 days	ROS; lipid peroxidation; lysosomal stability; DNA damage; phagocytosis	Cr(VI) induced oxidative stress, lysosomal destabilisation, oxidative DNA damage, lipofuscin accumulation, and reduced phagocytic activity.	[48]
Selenate and selenite	*E. andrei*	Whole-animal exposure to different Se forms and concentrations	Acute toxicity; apoptosis; efflux pump activity; oxidative stress; gene expression	Selenite caused greater acute toxicity, while selenate accumulated more strongly and induced apoptotic-like coelomocyte death. Both Se forms altered oxidative status.	[49]
Cu and Cd	*E. hortensis*	Isolated coelomocytes; in vitro; 32–250 µM	ROS production	Cu and Cd induced ROS production, which was reduced by EDTA, indicating metal-ion-mediated oxidative stress.	[50]
Cement kiln dusts	*L. terrestris*	In vitro: 10–500 mg L^−1^, 18 h; in vivo soil: 10–1000 mg kg^−1^	Viability; phagocytosis; flow cytometry	In vitro exposure reduced viability and phagocytosis, whereas soil exposure showed high variability and weaker effects, highlighting the importance of exposure route.	[51]

**Table 4:** Effects of organic pollutants, nanomaterials, and plastic particles on earthworm coelomocytes and coelomic fluid, with emphasis on oxidative stress, cytotoxicity, and immune impairment.

Pollutant	Species	Exposure Design	Main Endpoints	Key Coelomocyte/Coelomic Fluid Effects and Interpretation	Ref.
Chlorothalonil	*E. fetida*	Soil exposure; 18.5 and 37.0 mg kg^−1^; 14 days	Metabolomic changes in whole earthworms, coelomic fluid, and coelomocyte extracts	Coelomic fluid was the most sensitive matrix. Increased glutamine was detected at both doses, while N-acetylserine and ophthalmic acid increased at the higher dose, suggesting oxidative stress-related metabolic perturbation.	[52]
Pentachlorophenol (PCP)	*E. andrei*	Aged polluted soil; 15 and 150 ppm PCP; soil ageing for 20, 60, or 120 days; earthworm exposure for 7 or 14 days	Lysosomal membrane stability; coelomocyte subpopulation distribution; mortality; bioavailability	Lysosomal membrane stability was reduced in all treatments. Coelomocyte subpopulation changes occurred mainly in 60- and 120-day aged soils at 150 ppm PCP, while mortality increased in aged soils spiked with 150 ppm PCP.	[53]
7,12-Dimethylbenz[*a*]anthracene (DMBA)	*E. hortensis*	Isolated coelomocytes; in vitro exposure; 20–400 µM for ROS production and 50–200 µM for phagocytosis assay	ROS production; phagocytic activity	DMBA induced ROS production and reduced phagocytosis of hyaline amoebocytes, indicating oxidative stress and immunosuppressive effects on cellular immune function.	[50]
Functional nanocarbon black (FNCB) and FNCB + Cd mixtures	*E. fetida* coelomocytes	Isolated immunocompetent coelomocytes; in vitro exposure to FNCB and FNCB + Cd mixtures; dose-dependent mixture analysis	Cytotoxicity; intracellular free Cd^2+^; mixture toxicity modelling	FNCB caused coelomocyte damage, likely related to oxygen-containing surface groups. In mixtures with Cd, effects were antagonistic at low doses but synergistic at high doses, reflecting altered intracellular Cd^2+^ availability and particle–metal interactions.	[54]
Polyethylene microplastics	*E. andrei*	Soil exposure to two microplastic sizes; 21 days	Coelomocyte viability; growth; spermatogenesis; nanoplastic generation	Microplastic exposure reduced coelomocyte viability and affected growth and male reproductive organs. Earthworms fragmented ingested microplastics into nanoplastics, which were released into soil through casts.	[55]

**Table 5:** Principal cellular immune mechanisms mediated by earthworm coelomocytes, including phagocytosis, brown body formation, rejection of foreign tissue, extracellular trap formation, and cytotoxic responses.

Cellular Immune Mechanism	Main Cells/Factors Involved	Mechanism	Biological Role	Ref.
Phagocytosis	Hyaline amoebocytes; opsonins	Engulfment and degradation of foreign particles	Elimination of pathogens and debris	[1,13]
Brown body formation	Amoebocytes, eleocytes/chloragogen-derived cells, phenoloxidase, melanin	Encapsulation and aggregation of foreign material	Isolation and degradation of pathogens	[11,64]
Rejection of foreign tissue	Coelomocytes; humoral enzymes	Encapsulation and cytotoxic destruction of grafts	Recognition and elimination of foreign tissue	[13,65]
Extracellular traps (ETs)	Coelomocytes, ROS, histones, proteases	Extrusion of DNA–protein networks	Immobilisation and killing of pathogens	[14]
Cytotoxic activity against tumour cells	Granular and agranular coelomocytes	Contact-mediated cytotoxicity and debris removal	Elimination of abnormal or tumour cells	[12,44]

**Table 6:** Major humoral immune factors identified in earthworms and their biological functions, mechanisms of action, and representative species in which they have been described.

Humoral Factor	Type	Main Activity/Function	Mechanism/Target	Species/Examples	Ref.
Lysenin	Pore-forming protein	Haemolytic and cytotoxic activity	Binds sphingomyelin and forms membrane pores	*E. fetida*, *E. andrei*	[15,68]
Lysenin-related proteins, including fetidin/LRP-2	Lysenin-related proteins	Haemolytic and cytolytic activity	Homologous to lysenin; fetidin is considered lysenin-related protein 2	*E. fetida*, *E. andrei*	[15]
Toll-like receptors (TLRs)	Pattern recognition receptors	Pathogen recognition and signalling activation	Recognition of conserved microbial structures	*E. andrei*, *D. veneta*	[71]
LBP/BPI proteins	LPS-binding proteins	Recognition of Gram-negative bacteria	Binding and transport of lipopolysaccharides	*E. andrei*	[62]
Coelomic cytolytic factor-1 (CCF-1)	Pattern recognition receptor/opsonin	Broad-spectrum microbial recognition; activation of prophenoloxidase cascade	Binds LPS, β-1,3-glucans, and microbial carbohydrates	*Eisenia* spp., *L. terrestris*, *D. veneta*	[13,15]
Serine proteases	Enzymes	Activation of pro-PO cascade and coagulation	Proteolytic activation of immune pathways	*E. fetida*, *L. terrestris*	[61]
Perforin	Cytotoxic pore-forming protein	Induction of apoptosis in target cells	Forms membrane pores enabling protease entry	Earthworm coelomocytes	[44,72]
Cellulase	Hydrolytic enzyme	Cellulose degradation; possible immune defence role	Cleavage of β-1,4-glycosidic bonds	*E. andrei*	[73]
Lysozyme	Antimicrobial peptide/protein	Antibacterial and antiviral activity	Hydrolysis of bacterial cell wall peptidoglycan	*E. fetida*	[13,63]
Lumbricin I	Antimicrobial peptide	Broad-spectrum antimicrobial activity	AMP-mediated microbial killing	*L. rubellus*	[26]
PP-1	Antimicrobial peptide	Mucosal defence	Homologous to lumbricin I	*P. tschiliensis*	[13]
OEP3121	Antimicrobial peptide	Unknown	Poorly characterised AMP	*E. fetida*	[13]

## Data Availability

The data supporting the findings of this study are available from the corresponding author upon reasonable request.
